# The expression of Wnt2b in the optic cup lip requires a border between the pigmented and nonpigmented epithelium

**Published:** 2010-12-14

**Authors:** Junko Kitamoto, Jeanette Hyer

**Affiliations:** Department of Ophthalmology and Neurosurgery, University of California, San Francisco

## Abstract

**Purpose:**

Wnt2b is normally expressed at the optic cup lip and is implicated in ciliary body induction. The lens has often been considered an organizer for the anterior eye, but recent studies demonstrate that the anterior cell fates are correctly specified in the absence of the lens. This study uses Wnt2b as a marker to reveal the mechanism behind the specification of the anterior domain of the optic cup.

**Methods:**

Developing chick embryos were used as a model system. Eyes were microsurgically manipulated to assess the role of the lens in the development of the anterior optic cup. Eyes were molecularly manipulated, using fibroblast growth factor expressing replication-incompetent retrovirus, introduced into the retinal pigmented epithelium (RPE) domain. Ectopic fibroblast growth factor transformed the RPE into nonpigmented epithelium (NPE; ciliary body). As the virus does not spread, discrete borders between RPE and NPE were experimentally created. Wnt2b expression was assessed after surgical and molecular manipulation.

**Results:**

Contrary to expectations, we found that the lens is not able to induce Wnt2b expression in optic cup tissue: When the optic cup lip is experimentally misspecified such that it no longer contains the juxtaposition of pigmented and nonpigmented tissue, Wnt2b is not expressed. In addition, if the prelens ectoderm is removed from the optic vesicle before morphogenesis, the resulting lensless optic cup expresses Wnt2b even though it was not in contact with lens tissue. We also show that ectopic lenses do not induce Wnt2b in optic cup tissue. The ciliary body/anterior eye domain is specified at the border of RPE and the NPE of the ciliary body. During development, this border is normally found at the optic cup lip. We can manipulate tissue specification using retroviral-mediated gene transfer, and create ectopic borders between nonpigmented and pigmented tissue. At such borders, Wnt2b is ectopically expressed in the absence of lens contact. Finally, we describe a role for the lens in maintenance of Wnt2b expression and demonstrate support for this in two ways: First, we show that if the lens is removed from the formed optic cup, endogenous Wnt2b expression is specifically lost from the optic cup lip; and second, we show that while ectopic Wnt2b expression is initially found in the majority of ectopic borders, as eye development proceeds ectopic expression is maintained only in those borders that are close to the lens.

**Conclusions:**

Taken together, the results provide support for a model in which the anterior optic cup domain, as described in part by Wnt2b expression, is specified through the elaboration of a border within the optic neuroepithelium rather than through interactions with the surrounding environment.

## Introduction

A central question in development is how very different tissues arise from a common embryonic tissue precursor. In the eye, this question is strikingly presented, as the optic neuroepithelium differentiates not only into neurons, but also into the nonneural retinal pigmented epithelium (RPE), the secretory tissue of the ciliary body, and the musculature of the iris. The future eye tissue is first identifiable as laterally expanding vesicles in the anterior neural tube. The optic vesicles make contact with the overlying surface ectoderm and invaginate to form double-layered optic cups. The outer layer assumes the fate of pigmented epithelium, a nonneural support tissue, and the inner layer becomes the sensory neural retina, which contains several different types of specialized neurons. In the anterior portion of the eye, the sensory neural retina tissue abruptly ends at the ora serrata, and the inner layer continues as a nonneural, nonpigmented epithelium (NPE) into the ciliary body. As it continues into the iris, the inner layer also becomes pigmented. The pigmented epithelium (RPE) of the outer layer extends anteriorly without a dramatic change in appearance. However, in the ciliary body, the RPE becomes specialized for the secretion of aqueous humor and in the iris it expresses muscle proteins and forms the dilator muscle of the pupil.

The impact of the environment around the forming eye is substantial. The neural retina domain is committed first, through a physical interaction between the distal tip of the optic vesicle and the overlying surface ectoderm [1,2]. The RPE, in contrast, commits slowly. RPE’s fate depends on interaction with the surrounding mesenchyme [3–5] and maintained expression of the Micropthalmia-associated transcription factor (MiTF) and vertebrate orthodenticle homolog (Otx) transcription factors [6–8]. The RPE can be induced to form neural retina if exposed to fibroblast growth factor (FGF) signaling [1,9–13].

The differentiation of tissues of the anterior optic cup, the ciliary body, and iris, is less well understood. The refinement of several gene patterns, such as *Otx1*, vertebrate paired homolog 6 (*Pax6)*, bone morphogenetic protein 4 (*Bmp4)*, bone morphogenetic protein 7 (*Bmp7)*, from large expression domains to discrete optic cup lip expression domains, mirrors the observation that anterior eye fates are slowly imposed onto the anterior of the optic cup [14–18]. The lens has a role in specifying some aspects of the anterior eye, as ectopic lenses can induce some anterior specific genes [19]. It has also been demonstrated, using the blind cavefish, that replacing a mutant lens with a normal one completely rescued eye development [20]. Recently, however, Zhang et al. [21] used a genetically controlled toxin to delete the prelens ectoderm in mouse, to show that the early specification of the iris and ciliary body occurred in the complete absence of the lens; this corroborates similar observations we have made in the developing chick eye. If the role of the lens in the early specification of anterior optic cup fates is not as central as previously thought, then new mechanisms for the specification of anterior optic cup fates must be considered.

One potential mechanism for inducing the anterior portion of the optic cup is through Wnt signaling. Wnt signaling is active in the anterior optic cup, as demonstrated by β-catenin signal reporter constructs [22–24]. Several Wnt ligands are expressed in the anterior optic cup in the chick, including 2, 5a, 6,7a, 9a, and 16 [24–26]. However, of these, the only one expressed early in optic cup formation and in both chick and mouse eye development is Wnt2b, making it the most likely candidate [27]. Wnt2b is highly expressed in the optic cup lip through several stages of eye development, making it an excellent marker of the most anterior fate of the optic cup [28]. In addition, Wnt2b induces collagen IX, a specific marker of the functional ciliary body [29]. Therefore, in this study we focus on how Wnt2b expression in the optic cup lip is regulated in an effort to understand how the anterior optic cup is established.

The development of the optic cup was manipulated in several ways. The instructive environment of the anterior optic cup was disrupted by surgically removing the lens. The results reveal that the lens is not required for the expression of Wnt2b in the optic cup lip, but is required for maintenance of expression levels. We also experimentally created a linear “optic cup lip,” where RPE and NPE are arranged in a continuum, as seen at the hinge of the optic cup. Ectopic linear optic cup lips express Wnt2b without lens involvement. Finally, we observed the temporal refinement of Wnt2b expression to the ectopic borders between pigmented and nonpigmented tissue, and the lens-influenced maintenance of that expression. In total, we provide evidence that clarifies the role of the lens in eye development and points to distinct mechanisms involved in the development of the anterior eye tissues.

## Methods

Fertile chick eggs were supplied by Petaluma Farms (Petaluma, CA). Incubators were supplied by Georgia Quail Farm (Savannah, GA). Eggs were incubated until the desired embryonic stages as indicated in the text. Eggs were not developed past embryonic day 10.

### Retroviral injection

The retrovirus used in this experiment is a replication-defective variant based on the avian spleen necrosis virus and coexpresses FGF and β-galactosidase. Construction of the viral vectors, biologic activities of the FGF construct, and the performance of microinjection have been described previously [21,30–32]. Briefly, virus-containing supernatant was collected from packaging cells and the retroviral particles were concentrated by centrifugation at 15,000× g, 25 °C for 1.5 h. Virus-containing pellets were resuspended in the smallest possible volume and polybrene (hexadimethrine bromide, Sigma, St. Louis, MO) added to a final concentration of 100 µg/ml in virus solution. Virus particles were loaded into a pulled glass needle, directed to the desired tissue with the aid of a micromanipulator, and pressure injected (Harvard Apparatus Injector, model PLI-100, Holliston MA) into the optic vesicles of Hamburger-Hamilton (HH) stage 10–12 chicken embryos [33]. After injection, the eggs were resealed with Parafilm (Pechiney Plastics, Chicago, IL) and incubated at 37 °C for an additional 3 to 4 days until reaching the HH stage indicated.

### Microsurgical techniques

Lensless optic cups: Microsurgical techniques to make lensless optic cups have been well described elsewhere [34]. Briefly, the microsurgery was performed on carefully staged 16 or 17 somite embryos (HH stage12+/13-). The lens placode and surface ectoderm overlying the optic vesicles were stained with approximately 1 μl of 1.5% (W/V in water) Nile blue sulfate (Sigma), applied directly to the surface ectoderm with a pulled small glass needle. Nile blue is a vital dye used to detect apoptotic nuclei in whole embryos. Here, the application of Nile blue had two purposes: First, it stained the overlying surface ectoderm for identification and evaluation of removal and second, at the concentration used, it caused the ectoderm to lift off the underlying tissues, facilitating its removal without staining or disturbing the underlying tissue [34]. After surgery, the eggs were resealed with Parafilm (Pechiney Plastics) and incubated at 37 °C for an additional 24 or 48 h, as indicated.

Lens-extirpation: Embryonic eyes were exposed and cuts were made in the overlying surface ectoderm using sharpened tungsten needles. The ectoderm was pulled to the side and the zonules holding the lens were carefully teased away with sharpened needles until the lens could be lifted free of the optic cup. After surgery, the eggs were resealed with Parafilm (Pechiney Plastics) and incubated at 37 °C for an additional 24 to 48 h as indicated.

Lens implantation and culture: Host HH stage 23 optic cups were dissected clear of the embryonic head and placed in ice-cold Tyrode’s saline. Donor lenses were dissected clear of the appropriately staged heads and placed in ice-cold Tyrode’s saline. Dissected optic cups were held in place with small ring-shaped stages (hand-made) and tungsten needles were used to make openings in the RPE. Ball-tipped glass needles were used to gently tease apart the RPE and neural retina (NR) without additional tearing, and donor lenses were eased into position under the RPE. Specimens were placed in culture media Dulbecco's Modified Eagles Medium (DMEM/H-21; 4.5 g/l glucose, 0.5 g/l L-glutamine, 3.7 g/l sodium bicarbonate, Cell Culture Facility, UCSF, San Francisco CA), 10% fetal bovine serum, 1% chick embryo extract) and incubated at 37 °C/5% CO_2_ for 48 h.

### In situ hybridization

A plasmid containing about a 1 kb fragment of chick Wnt2b was kindly provided by Dr. J.C. Belmonte (Salk Institute, La Jolla, CA). The chick collagen IX probe was provided by Dr. David Beebe (Washington University, Saint Louis, MO). The Lef1 probe (Lymphoid enhancer binding protein factor 1) was provided by Dr. Mineko Kengaku (RIKEN Brain Science Institute, Wako City, Japan) [35,36]. The antisense riboprobes were synthesized in a reaction mixture containing digoxigenin-labeled UTP (Roche, Germany) and RNA polymerase (Promega, Fitchburg, WI) according to manufacturer's protocol.

Chick heads and whole eyes were fixed in 4% of paraformaldehyde in PBS for 2 h at room temperature or overnight at 4 °C. For embryos older than stage 22, the eye was dissected clear of surrounding tissue and the lens was removed. After several rinses in PBS, the specimens were incubated in 30% sucrose until equilibrated, embedded in a mixture of optimal cutting temperature (OCT) compound (Tissue-Tek)/10% sucrose and cryosectioned at 10–14 μm of thickness. For whole mount in situ hybridization, the specimens were dehydrated in methanol. In situ hybridization was performed using a modified protocol [37]. Briefly, the slides were dried completely, refixed in 4% paraformaldehyde, incubated in 20 μg/μl of proteinase K, refixed. The sections were acetylated for 20 min with 0.25% (v/v) acetic anhydride in 0.1 M triethanolamine, pH 8.0. Prehybridization at 65 °C for 2 h was done before adding 1 μg/μl of cRNA probes to hybridization solution (50% formamide, 5× SSC (0.75 M NaCl, 0.075 M Sodium Citrate), 1% Roche blocking regent, 5 mM EDTA, 100 μg/μl yeast tRNA (tRNA), 0.1% CHAPS, 0.1% heparin). Posthybridization washes were done in SSC/formamide solution in 65 °C. The specimens were blocked in 1% Roche blocking reagent (Roche) and incubated with alkaline phosphatase–labeled antidigoxigenin antibody (Roche) at 4 °C overnight. The color development was performed using nitro blue tetrazolium/5-bromo-4-chloro-3-indolyl phosphate (NBT/BCIP; Roche) after several washings with Tris-buffered saline, 0.1% Triton X-100 (TBST) and incubation in NTMT (Tris buffer, 0.1 M NaCl, 0.08 M MgCl_2_, 0.1% Tween-20). For whole mount in situ the same protocol was used, but the prehybridization step was increased to overnight.

### Immunohistochemistry

The sections were prepared in the same way as in situ hybridization. After drying, the sections were fixed in –20 °C methanol, washed in PBS solution, and blocked in 1% BSA (BSA) and 0.1% Triton X-100 in PBS for at least 30 min at room temperature. Antibodies against chick collagen IX (clone 2C2), chick anti-Na,K-ATPase (a5) and chick Pax6 (clone Pax6) were used at concentrations of 1:100, 1:800, and 1:150, respectively. These antibodies were obtained through the Developmental Studies Hybridoma Bank. Antibody against chick δ-crystallin was a kind gift from Dr. Joram Piatigorsky (National Eye Institute, Rockville, MD). Antibody against rat connexin43 (BD Transduction Labs) was used at a dilution of 1:800. Secondary antibodies were Alexa Fluor dyes (Molecular Proves). Antimouse IgG1 Alexa Fluor 594 was used for the detection of collagen IX, Na,K-ATPase, connexin43, and Pax6. Vectashield with 4',6-diamidino-2-phenylindole (DAPI; Vector Laboratories, Burlingame, CA) was used for mounting and staining of nuclei. Images were captured by a SPOT camera (SPOT Imaging Solutions, Sterling, MI) attached onto a Nikon Eclipse E800 (Nikon).

## Results

### Wnt2b is a marker of the anterior optic cup

**Figure 1:**
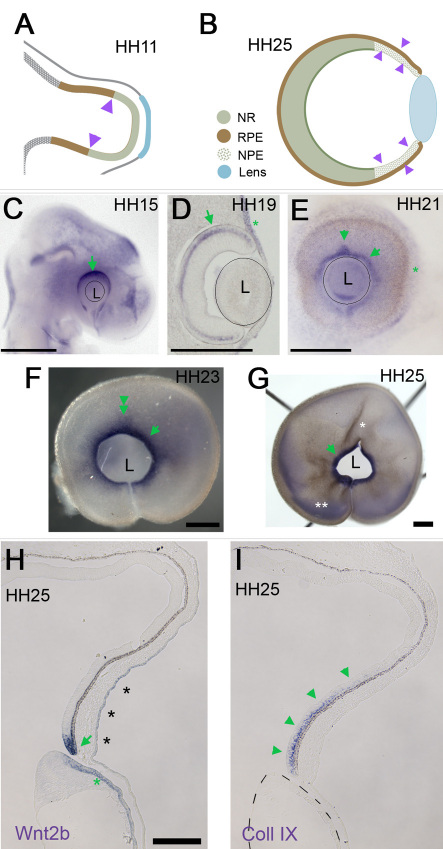
Wnt2b is a marker of the anterior optic cup lip. **A**: A graphic of the chick optic vesicle at Hamburger-Hamilton (HH) stage 11 (embryonic day 1.5) in which the presumptive neural retina (NR) is indicated in green and the presumptive retinal pigment epithelium (RPE) in brown and purple arrowheads mark the presumptive ciliary body/iris. **B**: A graphic of the optic cup, formed after invagination of the optic vesicle. At HH stage 25 (embryonic day 5), the ciliary body/iris is molecularly identifiable (light green dots, purple arrowheads). **C**-**G**: Whole mount in situ hybridization for Wnt2b gene expression through various stages of eye development. **C**: At HH stage 15 (embryonic day 2), a few hours after the initiation of invagination, Wnt2b expression is enriched in the dorsal optic tissue (arrow). **D**: Sections through an HH stage 19 embryo (embryonic day 2.5) showing Wnt2b expression throughout the RPE (arrow) and the overlying surface ectoderm (asterisk). The lens is outlined. **E**: At HH stage 21 (embryonic day 3) Wnt2b is refined to the anterior optic cup lip (arrowheads). Expression is also found throughout the surface ectoderm (asterisk). **F**: At HH stage 23 (embryonic day 4) Wnt2b expression is robust in the optic cup lip, with faint expression in RPE. **G**: At HH stage 25 (embryonic day 5) Wnt2b is expressed exclusively in the optic cup lip (arrow); white asterisk indicates a rip artifact, double white asterisk indicates non-specific background staining. **H**: Section in situ analysis confirms the restriction of Wnt2b signal (green arrow) to optic cup lip at HH stage 25. Brown is the endogenous color of the RPE. The lens epithelium (green asterisk) and the surface ectoderm (black asterisk) also express Wnt2b. **I**: Adjacent section stained for collagen IX shows the extent of specified ciliary body tissue (green arrowheads) in the anterior of the optic cup. Dashes outline the lens. Scale bars in **C**-**G** are equal to 500 µm, and in **H** are equal to 100 µm. Abbreviations used are as follows: L-lens; staging table; NPE-non-pigmented epithelium of the ciliary body.

In the chick, the eye develops from an optic vesicle into a fully patterned optic cup over the course of three days. Portions of the future optic cup can be detected at optic vesicle stages; the future neural retina and RPE are molecularly identifiable at HH stage 11 (Figure 1A,B) [33]. In the anterior of the optic cup, the future ciliary body can be identified before it is morphologically distinct, as it expresses collagen IX, a vitreous protein that is exclusively expressed from the mature ciliary body [38]. It is not clear whether the anterior domain is specified at optic vesicle stages, as collagen IX is not detectable until after the optic cup has formed.

Wnt2b is a particular marker for the most anterior portion of the optic cup, the hinge region where the RPE doubles back on itself to form the inner layer of the optic cup [28]. Expression during eye development reveals that this pattern is a refinement of widespread RPE expression (Figure 1C-G). Wnt2b is never expressed in the inner layer of the optic cup, and we did not detect it at the optic vesicle stages (data not shown). Although, as described, Wnt2b has a potentially important role in maintaining the progenitor populations for continued eye growth, to date it is not understood how Wnt2b is regulated. Here, we look at the requirements for the refined pattern at HH stage 25, where Wnt2b is expressed precisely at the optic cup lip (Figure 1H,I).

### Wnt2b expression in the optic cup lip does not depend on lens tissue

We used an FGF-expressing replication-incompetent retrovirus to create sources of ectopic FGF signal within the developing eye. The cloning and validation of the FGF-expressing retroviral vectors in this study, including control viruses, have been extensively described in prior publications [1,21,39].

Because all of the viral structural genes have been removed from the viral vector, the virus cannot propagate itself and is produced using a packaging cell line that supplies those proteins in cis [31,32]. Infection is limited to those cells exposed to virus-containing solution at the time of introduction. The replication-incompetent retrovirus used throughout the experiments described here coexpresses human FGF4 and β-galactosidase. The infected cells and their direct progeny can be followed by localizing β-galactosidase with X-gal staining.

**Figure 2:**
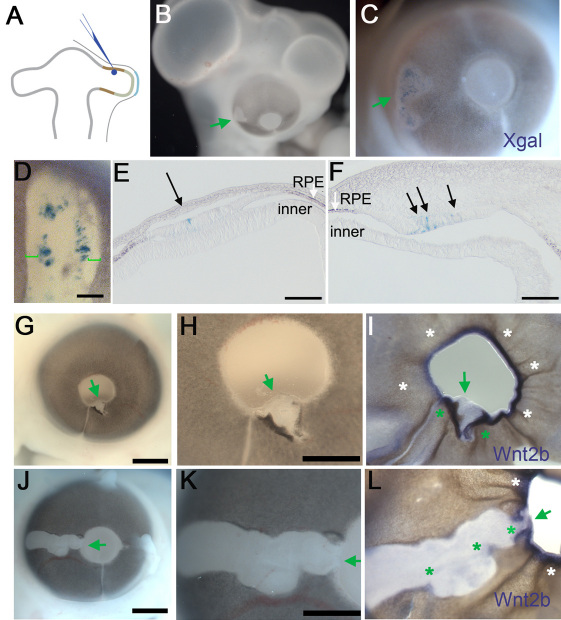
Wnt2b is not expressed at optic cup lip if anterior pigment epithelium (PE) is mis-specified. **A**: A Graphic of the introduction of replication-incompetent retrovirus into the presumptive retinal pigment epithelium (RPE) at optic vesicle stages. **B**: An embryo at Hamburger-Hamilton (HH) stage 25, 4 days after injection. White patches are seen on the infected eye. **C**: The eye of embryo in **B**, stained with X-gal (blue stain) to visualize infected cells within the depigmented patch (green arrows). **D**: X-gal stained eye, showing that a white depigmented zone consistently extends beyond immediately vicinity of infected cells (green brackets). **E**, **F**: Section analysis through additional white patches, showing outer RPE (brown pigmentation, white arrows), a depigmented portion contiguous with the RPE (black arrows) and the inner layer of the optic cup (as labeled). Infected cells (blue cells, black arrowheads) are found in the RPE layer. At a distance of approximately 100 microns from infected cells the depigmented tissue transitions back into RPE tissue. **G**-**L**: Infected eyes at HH stage 25 with depigmented patches that fall in the anterior optic cup lip (arrows). **G**, **H**: An eye with a small patch of depigmentation at the extreme edge of the optic cup (arrow). **I**: The same eye as in **H** after in situ hybridization to localize Wnt2b expression showing no expression in the depigmented optic cup lip (arrow). All parts of the unaffected optic cup lip express Wnt2b in a lip specific pattern (white asterisks). At the transition zones occurring at the edges of the depigmented patch, Wnt2b is ectopically expressed (green asterisks). **J**, **K**: An eye with a larger patch of mis-specified anterior optic cup lip (arrow). **L**: The eye of embryo in **K** showing Wnt2b gene expression. No expression is found in mis-specified lip (arrow). Unaffected regions of the eye express Wnt2b at the lip (white asterisks). Aberrant Wnt2b expression is found at the transition zone between pigmented and depigmented tissue. Scale bars in **D**-**F** are equal to 200 µm, in **E** and **G** are equal to 500 µm and in **H** and **K** are equal to 250 µm.

The FGF-expressing virus was targeted to the pigmented epithelium domain of the HH stage 10 chick optic vesicle (Figure 2A). The embryos were resealed and incubated for a further four days, during which the optic vesicle developed into an optic cup (as in Figure 1A,B). Eyes that were infected at the optic vesicle stages exhibited white patches (Figure 2B). X-gal staining revealed that the white patches contained infected β-galactosidase-expressing cells (Figure 2C-F). The infected cells coexpress FGF4, and this affects the neighboring noninfected cells. The control virus, which only expresses β-galactosidase, did not produce this effect (n=75; data not shown [1]). Sections through the white patches showed that infected (blue) cells lie within the RPE layer and influence the phenotype of neighboring cells for up to 100 μm from the site of the ectopic FGF source (Figure 2D-F). Distance from infected cell to pigmented epithelium is 211±82 μm, n=41). Depigmentation from infection was seen throughout the RPE, in both the anterior hemisphere near the lens and in the posterior hemisphere, at the back of the eye. Transition zones occurred at the edges of the patches, where the normal RPE transitioned into a depigmented epithelium. The depigmented portion is identifiable as the NPE of the ciliary body, as it expresses collagen IX, connexin43, Nidogen, and Laminin, all markers of the functional ciliary body [21]. Therefore, at this transition zone, the RPE was misspecified as a ciliary body by exposure to distally produced FGF4.

By targeting the virus to the presumptive optic cup lip at the vesicle stages (Figure 1A), eyes were produced with white patches that involved the lip of the optic cup (Figure 2G-L). These “lens-patch” eyes were probed for Wnt2b expression, a marker for the cells that make up the hinge/lip of the optic cup at the stages examined (HH stage 25, Figure 1). The affected tissue around infected cells was consistently discrete and unaffected/uninfected portions of the eye served as internal controls for expression levels. We found that Wnt2b is not expressed as expected at the hinge/lip portion of any given lens-patch (Figure 2I,L, green arrows; n=15). Rather, Wnt2b was aberrantly expressed in transition zones at the edges of the white patches (green asterisks, Figure 2I,L).

**Figure 3:**
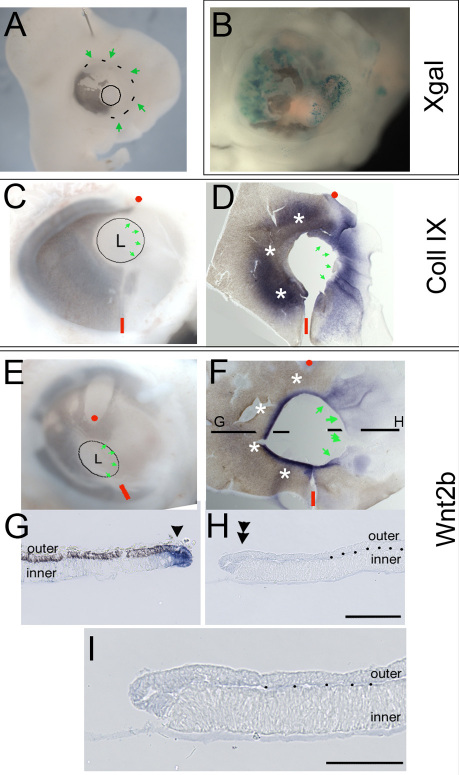
The lens cannot induce Wnt2b expression in anterior optic cup lip. **A**: A highly infected eye with a large patch such that the entire nasal hemisphere (green arrows) is depigmented. **B**: A second highly infected eye after Xgal staining. **C**: Similarly infected eye at Hamburger-Hamilton (HH) stage 25, unstained and **D**: after in situ hybridization to localize collagen IX expression. A red dot and dash orient between the two panels. CollagenIX expression is found in the depigmented optic cup lip (green arrows in **D**). White asterisks indicate collagen IX expression in the unaffected anterior, which serves as a native internal control. **E**: A highly infected eye at HH stage 25, unstained and **F**: after in situ hybridization to localize Wnt2b expression. A red dot and dash orient between the two panels, white asterisks indicate native Wnt2b expression in the unaffected anterior cup lip and green arrows indicate lack of Wnt2b expression in the depigmented lip. The section plane position for panels **G**-**I** are indicated. **G**: A section through unaffected optic cup lip shows endogenous expression of Wnt2b (arrow) at the hinge between outer retinal pigment epithelium (RPE) layer and inner ciliary body tissue. **H**,**I**: A section through the mis-specified optic cup lip, showing no Wnt2b expression in the hinge (double arrowheads). The outer layer is completely depigmented and dots demarcate the border between the two epithelia. Scale bars are equal to 50 µm.

We next examined whether the lack of Wnt2b expression in the depigmented optic cup lip was specific to the regulation of Wnt2b or a result of generalized misspecification of the anterior tissue. We used collagen IX as a reliable marker of specified ciliary body tissue. However, collagen IX is also induced in transition zones at the edges of white patches [21]. On average, this induced collagen IX expression extends 100 μm from any pigmented tissue into adjacent depigmented tissue (n=6, as measured in whole mount views of transition zones; data not shown). Therefore, to examine whether or not the anterior optic cup had been correctly specified, we created massively infected eyes, in which 50% of the anterior optic cup lip was depigmented (Figure 3A,B; n=6). Analysis revealed that the depigmented optic cup expressed collagen IX, similarly to the unaffected portion of the eye, which served as an internal control (Figure 3D, green arrows compared to white asterisks). The affected lip was correctly specified as ciliary body tissue, even though the normal organization of outer layer/RPE and inner layer/NPE were disrupted (Figure 3C,D). In contrast, analysis of Wnt2b expression revealed that the depigmented portion of the optic cup lip did not have any detectable expression, while the unaffected/internal control portion had characteristically robust expression of Wnt2b at the lip (Figure 3F, green arrows compared to white asterisks). These findings were confirmed by section analysis (Figure 3G-I).

**Figure 4:**
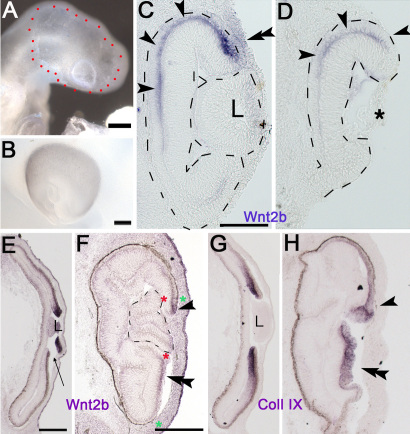
Wnt2b expression is not dependent on presence of lens tissue. **A**: An example of the pre-lens ectoderm removal surgery with red dots indicating the extent of ectoderm removal. **B**: The operated eye 48 h after re-incubation. **C**: The contralateral control eye and **D**: lens-less operated eye stained for Wnt2b expression 24 h after surgery. Note that in these sections the eye flattened and the vitreous space is not obvious. Expression is found in the outer layer of the anterior optic cup (single arrowheads) and is more robust in the optic cup lip in the control eye (double arrowheads). An asterisk indicates non-lens tissue. **E**: Contralateral control and **F**: lens-less operated eye, 48 h after surgery. The lens-less eye is smaller and the neural retina has formed folds that occupy the center (dashed lines). Wnt2b gene expression is present at the junction of pigmented (green asterisk) and non-pigmented (red asterisk) tissue but reduced (overstained section). A typical-looking optic cup lip, with normal hinge formation and Wnt2b expression is indicated by the single arrowhead and double arrowheads indicate expression without hinge formation. In **E**, arrow indicates an artifact rip in the section. **G**: Contralateral control and **H** lens-less eye of embryo stained for collagen IX gene expression. **G** and **H** are sections adjacent to **E** and **F**. Expression is found in same domains where Wnt2b was seen. Scale bars in **A** and **B** are equal to 250 µm, and in **C**, **E**, **F** are equal to 200 µm.

Together, these findings imply that the lens does not initiate Wnt2b expression in the optic cup lip. In addition, we had observed (Figure 2) that Wnt2b was ectopically expressed in some of the transition zones at the edge of white patches (green asterisks, Figure 2I,L), implying that the Wnt2b expression was lens-independent. To test this, we removed the prelens ectoderm, before lens placode formation, but after neural retina specification, as described in previous work from the laboratory (Figure 4A; n=12 [34]). Under these conditions, the optic vesicle undergoes the typical morphogenetic transition to an optic cup, without concomitant lens development (Figure 4B). Potential lens reformation is assessed with δ-crystallin staining, the major lens crystallin in avians [40]. We have shown that even when the ectoderm regrows over the forming optic cup, it fails to express Sox2 or Pax6, both early markers for committed lens ectoderm [34]. When examined 24 h after the surgery, the optic cup had formed on the operated side (Figure 4D). We had previously shown that collagen IX is expressed in lensless eyes [21], demonstrating that the ciliary body is specified in the absence of the lens. Lensless optic cups also express Wnt2b (Figure 4D), albeit at lower levels than the contralateral control eye (Figure 4C).

We noted that the Wnt2b expression was not upregulated in the lip of the surgical eye as it is in the control eye. To determine whether Wnt2b is eventually refined in the lensless optic cup, we looked at later postsurgical time points. After leaving the eyes to develop for 48 h after surgery, it was found that the optic cup was smaller and the neural retina was folded and convoluted (Figure 4F; n=4. Note that the sections were overdeveloped). The folded appearance is similar to eyes in which the intraocular pressure is not established [41] and may result from an inability to create pressure without a lens in place. Wnt2b was expressed in these lensless optic cups, at lower levels than in the contralateral control (Figure 4E). Expression was seen in portions that could be easily identified as optic cup lips, with associated hinge regions (Figure 4F single arrowhead) and in portions that were areas of juxtaposed RPE and NPE, but without hinge-like structures (Figure 4F double arrowheads). An adjacent section stained for collagen IX expression confirmed the specification of anterior tissue (Figure 4G,H). Wnt2b, therefore, is refined to the anterior optic cup lip independently of the lens.

### Wnt2b is expressed in transition zones found at the edges of FGF induced patches

In the lensless optic cup experiments, Wnt2b expression levels in the operated eye did not approach the strength of expression in the contralateral control eye. Interestingly, as seen in large depigmented patches (example of Figure 2L), transition zones nearer to the lens had stronger ectopic Wnt2b expression than those at a distance from the lens. This indicated that the lens, or the generalized anterior environment, might have an effect on overall levels of expression.

**Figure 5:**
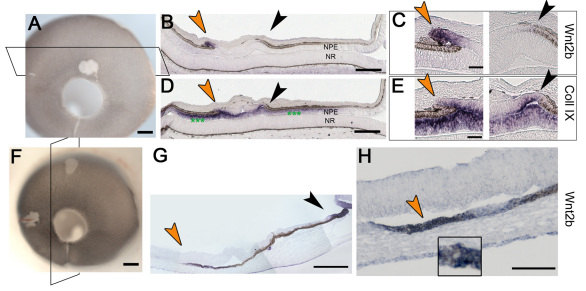
Wnt 2b is ectopically expressed in transition zones **A**: An infected eye at Hamburger-Hamilton (HH) stage 25 with virus associated depigmented patch at a distance of approximately 200 μm from lens. The section plane is indicated by a box. **B**-**E**: are sections through the eye in **A**. Note that in these sections the eye is flattened and the vitreous space is not obvious. The posterior neural retina (NR) lies directly in contact with the anterior epithelia (NPE, nonpigmented epithelium of the ciliary body; see Figure 1). **B**: Wnt2b gene expression is found at the edge of the patch; arrows bracket the patch of depigmented tissue with a transition zone at each edge. **C**: A Higher magnification view of portions of **B**, showing Wnt2b in one of the two transition zones (orange arrows) at the edges of white patch. **D**: Collagen IX expression in the section adjacent **B**; green asterisks the indicate endogenous expression in anterior epithelium (NPE). **E**: A higher magnification view showing collagen IX expression in both transition zones. **F**: An infected eye at HH stage 25 with a virus associated depigmented patch at a distance of approximately 1200 microns from lens. The section plane is indicated by a box. **G**: A section through the infected area and lens, showing endogenous Wnt2b signal in anterior optic cup lip (purple arrow) and ectopic Wnt2b signal at edge of infected patch (orange arrow); green asterisk indicates endogenous lens signal. **H**: A higher magnification of the transition zone in **G** (orange arrow). The inset panel shows the juxtaposition of Wnt2b expression and pigmented epithelium. Scale bars in **A** and **F** are equal to 500 µm, in **B** and **D** are equal to 200 µm, in **C**, E and **H** are equal to 100 µm and in **G** are equal to 300 µm.

To test this hypothesis, we examined additional transition zones around white/depigmented patches that were not associated with the optic cup lip and were found at a distance from the lens (Figure 5A,F). Sections were made through the affected patches and probed for Wnt2b and collagen IX expression.

In transition zones located within 500 μm of the optic cup lip (Figure 5A; n=4), Wnt2b was specifically expressed at the border between the pigmented and nonpigmented tissue (Figure 5B, orange arrow). Interestingly, in the same section, at the other side of the same patch, is a transition zone that does not express Wnt2b (Figure 5C, black arrow). An adjacent section probed for collagen IX expression (Figure 5D) reveals that both transitions zones are areas of ectopic ciliary body tissue, as both express collagen IX (Figure 5E). The anterior location of these particular transition zones is confirmed by the endogenous expression of collagen IX in the NPE of the inner layer (asterisks, Figure 5D). We next examined transition zones located greater than 500 μm from the optic cup lip (Figure 5F; n=7). In situ hybridization for Wnt2b expression also revealed a signal at the transition zone (Figure 5G, orange arrowhead). The signal was faint compared to endogenous expression at the optic cup lip (Figure 5G, black arrowhead), but present (Figure 5H).

**Figure 6:**
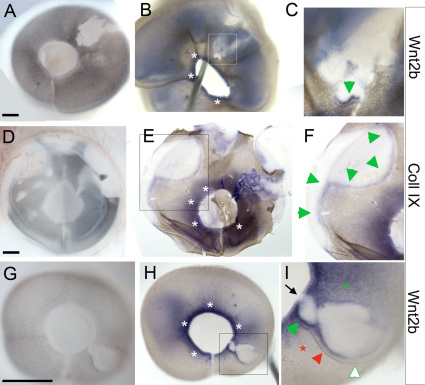
Wnt2b is preferentially maintained in anterior transition zones. **A**: An infected eye at Hamburger-Hamilton (HH) stage 25 with a virus associated depigmented patch. **B**: The same eye as in **A**, after in situ hybridization to localize Wnt2b expression. The white asterisks indicate endogenous Wnt2b expression in unaffected anterior optic cup lip. The boxed area is magnified in **C**. **C**: Only the transition zone closest to the lens expresses ectopic Wnt2b (green arrowhead). **D**,**E**: An infected eye at HH stage 25, before (**D**) and after (**E**) in situ hybridization to localize collagen IX expression. The white asterisks indicate endogenous collagen IX expression in unaffected anterior optic cup lip tissue. The boxed area is magnified in **F**. **F**: CollagenIX is ectopically expressed throughout the borders of the infected patch (green arrows). **G**, **H**: An infected eye at HH stage 23 before (**G**) and after (**H**) in situ hybridization to localize Wnt2b expression. The asterisks indicate endogenous expression, and the boxed area is magnified in **I**. **I**: At HH stage 23, Wnt2b is ectopically expressed throughout a large portion of the border area, with posterior borders less robust (white arrowhead) and anterior borders more robust (green arrowhead). A green asterisk indicates native expression in retinal pigment epithelium (RPE). Red asterisk indicates where RPE is no longer expressing Wnt2b. Transition zones in this area (red arrow) express Wnt2b. The black arrow indicates a depigmented patch at the lip that does not express Wnt2b. Scale bars in all panels are equal to 500 µm.

There did not appear to be a strict requirement of lens proximity for ectopic Wnt2b given these analyses. However, the data supported a conclusion that expression levels were more robust when located physically closer to the anterior optic cup/lens. To confirm the apparent role that lens proximity had on expression levels, we examined Wnt2b expression in isolated whole eyes (Figure 6). An eye was selected with a large depigmented patch, which created transition zones from within 500 μm of the lens, to distal points well over 500 μm along the edges of the posteriorly extending patch (Figure 6A; n=3). Wnt2b was appropriately expressed throughout the optic cup lip (Figure 6B, asterisks). In the affected patch, Wnt2b was unambiguously expressed only in the most anterior portion of the patch, and no other points along the edges had expression (Figure 6C, green arrowhead). In comparison, a similar eye was probed for collagen IX (Figure 6D,E; n=3), which was expressed at every point along the border of the patch (Figure 6F, green arrowheads).

In the experiments described above, the eyes were developed to HH stage 25, a point in development where endogenous Wnt2b expression is strictly expressed in the optic cup lip (Figure 1G). We next looked at an earlier stage of eye development, where Wnt2b is more widely expressed (HH stage 23; Figure 1F; n=4). Optic vesicles were infected with FGF-expressing retrovirus and removed after 3 days of reincubation. Embryos with depigmented patches, indicative of retrovirus infection (Figure 6G), were processed for Wnt2b expression. At these stages, endogenous Wnt2b was upregulated in the unaffected lip (Figure 6H, white asterisks). Expression was seen across the anterior RPE in unmanipulated portions. Surprisingly, upregulated expression was found in all portions of the border in the affected patch (Figure 6H). Ectopic Wnt2b was expressed in a gradient, lower in the posterior portions (Figure 6I, white arrows) and higher in the anterior portions closer to the lens (green arrow). Where endogenous Wnt2b expression was still widespread throughout the RPE (green asterisk), it was difficult to discern whether the increased signal was ectopic or not, but in those portions of the RPE no longer expressing (red asterisk), it was clear that expression was ectopic. In addition, portions of the transition zone had refined and upregulated expression reminiscent of the optic cup lip itself (red arrowhead). In the portion of the patch that involved the optic cup lip, no Wnt2b expression was found (black arrowhead), as had been observed in older eyes (Figure 2 and Figure 3).

### The lens maintains Wnt2b expression in the optic cup lip

Results from both HH stage 23 and 25 eyes reveal that Wnt2b is induced in all transition zones between the RPE and NPE/ciliary body, and is then refined to the anterior optic cup lip. Although we were able to show that the lens is not required for lip-specific expression (Figure 4), it was clear that the expression in the surgical eye never reached endogenous levels. To test whether the lens was instrumental in reinforcing and maintaining expression of Wnt2b at the lip, HH stage 21 and 23 embryos were exposed and the lenses were removed from the eye in ovo (n=4). Eggs were resealed and reincubated to HH stage 25 (24 and 48 h, respectively). As a control surgery, chick embryos were exposed and the overlying surface ectoderm and presumptive corneal ectoderm were removed from over the optic cup; the lens was left in place (n=6).

**Figure 7:**
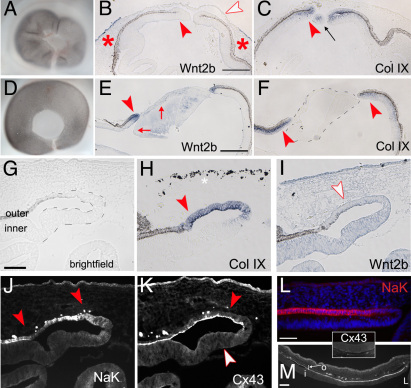
Without lens, endogenous Wnt2b expression is lost from anterior optic cup lip. **A**: A Whole mount view of the eye 24 h after lens removal. **B**: A section through eye in **A** showing loss of endogenous Wnt2b gene expression from lip (red-) and cornea (white-arrowheads). Signal is still retained in non-cornea surface ectoderm (red asterisk). **C**: An adjacent section showing collagen IX gene expression in lip (red arrowhead). Black arrow indicates a collagen IX positive piece of lip from a tissue fold, caught on section. **D**: Whole mount view 48 h after surface ectoderm removal. **E**: Section through the eye in **D**, showing retention of Wnt2b signal in lip (red arrowhead). The endogenous signal is lost from lens ectoderm (arrows). **F**: An adjacent section, shows collagen IX staining (red arrowhead). **G**-**H**: Adjacent section analysis of the post surgical anterior optic cup lip, 48 h after lens removal, as in **A**. **G**: A bright-field view of the post-surgical optic cup lip, shows that there is no lens. Dashes are used to outline the extent of tissue neuroepithellial tissue. The outer layer has endogenous pigmentation typical of the retinal pigment epithelium (RPE). This inner layer is contiguous with the neural retina. **H**: CollagenIX expression on an adjacent section (red arrowhead) is found in the outer layer of the optic cup lip after lens removal. The white asterisk marks non-signal. **I**: A section stained for Wnt2b expression, shows that Wnt2b is not found in the lens extirpated optic cup lip, even when overstained (white arrowhead). **J**: An adjacent section immunostained for the RPE marker NaK-ATPase (red arrowheads, see **L**) shows that the outer layer correctly expresses the RPE marker. **K**: The expression of the ciliary body marker connexin43. Cx43 is highly expressed in only in the outer layer of the anterior optic cup (red arrow) and is aberrantly absent from the inner layer (white arrowhead; see **M**). **L**: NaK-ATPase expression in control optic cup lip. Expression (Red signal) is found in RPE layer. **M**: Connexin43 expression in control optic cup lip. Expression (white signal) is found in a line between outer RPE layer (o) and inner ciliary body layer (i). L denotes lens. Inset box shows that the posterior neural retina does not express detectable levels of Cx43. Scale bars in **B** and **E** are equal to 200 µm, and in **G**, **L** and **M** are equal to 100 µm.

After lens removal surgery, the eyes appeared smaller and were slightly deflated (Figure 7A). Eyes in which the overlying surface ectoderm was removed appeared completely normal (Figure 7D). Sections were taken through eyes subjected to experimental manipulation and probed for gene expression. In lens-removed eyes, Wnt2b was not expressed in the optic cup lip. This effect was seen within 24 h of lens removal (red arrowhead, Figure 7B). Endogenous expression was also lost from the corneal ectoderm (white arrowhead). CollagenIX expression revealed that the optic cup lip did retain its identity as a ciliary body after the lens had been removed (Figure 7C). Removal of surface ectoderm did not affect the expression of Wnt2b in the lip. However, endogenous expression in the lens ectoderm was lost (red arrows, Figure 7E, compared to Figure 1H). We next examined the optic cup lip 48 h after lens removal, to confirm that the lip retained tissue specification (Figure 7G-K). Several parameters were assessed: pigmentation, collagen IX expression, sodium-potassium ATPase (NaK-ATPase, a marker of RPE [42]), and connexin43 (a marker of the ciliary body [43]) were all detected, confirming that the anterior optic cup lip remained correctly specified. No Wnt2b was expressed (Figure 7I). Some disorganization was apparent; for example, collagen IX was expressed in the outer layer of the postsurgical lip (Figure 7H). However, at present, this disorganization cannot be ascribed to loss of Wnt2b expression and may be related to the surgery.

### The lens does not induce de novo Wnt2b expression in optic cup tissue

**Figure 8:**
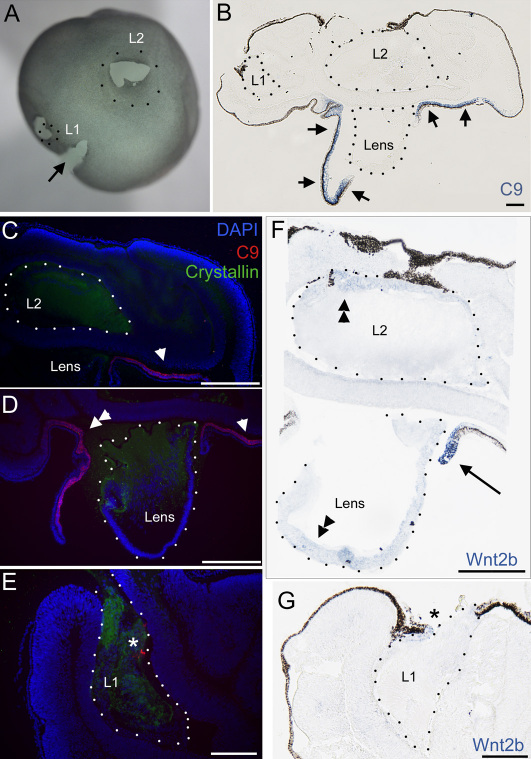
Ectopic lenses cannot induce Wnt2b or collagen IX gene expression in optic cup tissue. **A**: A whole mount posterior view of Hamburger-Hamilton (HH) stage 23 eye at the initiation of the experiment. Note that the endogenous lens is out of view. Lenses were placed under the retinal pigment epithelium (RPE) in the posterior of the eye. L1 indicates the position of a HH stage 15 implanted lens, L2 indicates the position of a HH stage 24 implanted lens and an arrow indicates the optic nerve. **B**: A section through the eye in A after 48 h in culture, and after in situ hybridization for collagen IX gene expression. The arrows indicate endogenous expression around the lens in the anterior of the eye and implanted lens are demarcated with dashes and labeled as in **A**. No collagen IX gene expression is seen around implanted lenses. The RPE tissue appears black and is broken at ectopic lens insertion points. **C**-**E**: Immuno-histochemistry for δ-crystallin and collagen IX expression on sections adjacent to those in **B**. Implanted lenses are demarcated with dots, and are positive for δ-crystallin (green signal). **C**: CollagenIX staining (red signal) is found in the endogenous anterior optic cup lip (arrowhead, indicates same position as arrowhead in **D**) and no signal is found associated with the ectopic lens, L2. **D**: CollagenIX expression (red signal, single and double arrowheads) is found in the anterior optic cup, in association with the native lens. **E**: CollagenIX protein (donor derived) is found associated with the HH stage 15 lens (asterisk, red signal), but is not found in host optic cup tissue. **F**: Wnt2b gene expression is shown in sections adjacent to those in **B**. The implanted lens L2 and the native lens have slight expression in lens ectoderm (double arrowheads). A strong Wnt2b signal is seen in the optic cup lip (arrow) but is not found in associated with implanted lens L2. Thickened areas of RPE are artifacts of growth in culture. **G**: Slight Wnt2b expression is found in the lens ectoderm of implanted lens L1 (asterisk), but is not found in the host optic tissue. Scale bars in **B**, **F**, and **G** are equal to 200 µm, and in **C**, **D,** and **E** are equal to 100 µm.

To further test whether the lens can induce Wnt2b expression, we implanted ectopic lenses into the optic cup. As described in Figure 1, Wnt2b expression is widespread early in development, and then is refined to the optic cup lip. Therefore, to assess de novo induction, we used eyes at HH stage 25, when Wnt2b is only expressed in the optic cup lip, and tested whether Wnt2b could be induced by ectopic lenses placed in contact with optic cup tissue. Donor lenses were either HH stage 15 or HH stage 24. The HH stage 15 was expected to be negative for inducing ability, as at this stage the lip-specific Wnt2b expression is not present (Figure 1C). In contrast, the HH stage 24 lens was expected to be positive for inducing ability (Figure 1H). The native lens served as an internal control for expression. Optic cups were carefully dissected away from the head and small incisions were made in the RPE. Donor lenses were carefully sandwiched between the RPE and NR in the posterior of the optic cup (n=5; Figure 8A), and the optic cups were cultured for 48 h. Resulting eyes were sectioned and examined for collagen IX and Wnt2b expression (Figure 8B-G). CollagenIX gene and protein expression and Wnt2b gene expression were found in the native optic cup lip (Figure 8B,D,F). No Wnt2b or collagen IX expression was seen around the implanted HH stage 15 lens (Figure 8B,E,G). The HH stage 24 lens did not induce any Wnt2b or collagen IX expression in the optic tissue with which it was in contact (Figure 8B,C,F).

## Discussion

The anterior hemisphere, containing the ciliary body and iris of the vertebrate eye, is anatomically complex and its development is not completely understood. Although it was classically thought that the lens was required to induce the ciliary body and iris from the optic neuroepithelium [19,20], other studies have demonstrated that the lens is not required for early induction and specification [21,44]. This study was undertaken to examine the role of the lens in controlling Wnt2b expression, the most anterior marker of the optic cup. As Wnt2b is expressed in the optic cup lip, that portion of the eye that is in direct contact with the lens, it was expected that the lens might directly induce expression.

Results from several experiments show that the lens cannot induce Wnt2b in optic cup tissue: 1) Wnt 2b is expressed in optic cups that have never been exposed to lens tissue (Figure 4F); 2) when the optic tissue next to the lens is misspecified such that the outer layer is depigmented, the lens cannot induce Wnt2b in that portion of the optic cup lip (Figure 2I and Figure 3F); 3) Wnt2b is ectopically expressed in portions of the eye that are not in contact with the lens (Figure 5C,H); and 4) ectopic lenses do not induce Wnt2b from optic cup tissue (Figure 8F,G).

Although Wnt2b expression in the anterior eye may be simplistically described as induced, it is more accurate to describe the changing expression pattern as a process of refinement to the anterior (Figure 1C-G). We were able to demonstrate that Wnt2b is refined not only to the tissue in direct contact with the lens, but also to tissue that defines a border between pigmented and nonpigmented optic epithelium (Figure 2I,L and Figure 4F). In the native eye, this border is the optic cup lip. In our experimentally created transition zones, a similar border could be created at will within the RPE tissue (Figure 5, Figure 6). Ectopic Wnt2b was refined to ectopic borders in a developmentally dependent manner, similar to the endogenous signal (Figure 6I).

Finally, we were able to demonstrate that the lens has a role in maintaining and perhaps enhancing existing Wnt2b expression. In the eyes that developed in the complete absence of a lens, Wnt2b expression was established and refined, but was clearly reduced in strength compared to its expression in control eyes. It is expected that if left to develop for an additional 24 h, expression would be completely lost; this experiment cannot be done, however, as the lensless eye does not grow and instead becomes more convoluted, eventually being overgrown by the head. In addition, the ectopic Wnt2b expression refined to ectopic borders is quickly lost; 24 h later, ectopic border Wnt2b is mainly found at those borders within 500 μm of the lens (Figure 5B, Figure 6C). Finally, native expression in the optic cup lip is lost if the lens is removed (Figure 7); even though the anterior optic cup lip retained the normal organization of RPE and NPE at the lip, it did not express Wnt2b. Although this is superficially confounding, it is understandable given the age of the eye during the analysis and the nature of the analysis: The majority of HH stage 25 eyes do not express ectopic Wnt2b at a distance from the lens, although it is possible (Figure 5G), and section analysis can sometimes miss very small areas of ectopic expression (Figure 5C, Figure 6C). The underlying concept for this experiment is that at the time of lens removal, the surgical eyes had already started to strongly express Wnt2b in the optic cup lip (Figure 1E,F), and that expression seems completely lost with the loss of the lens.

### The role of Wnt2b in the anterior optic cup

The specific role of the Wnt2b signal from the anterior has been investigated in several other studies [22,29,45,46]. The consensus conclusion is that Wnt2b/β-catenin signaling is involved in suppressing neurogenic genes and activating expression specific to the anterior optic cup, such as that of Msx1, Otx1, and collagen IX [29,45,47]. However, there are contrasting reports as to whether Wnt2b inhibits proliferation, a characteristic of the anterior tissue [22,45], or promotes proliferation as part of stem cell compartment signaling in the vertebrate eye [47,48].

Because Wnt2b is so strikingly expressed in the most anterior portion of the optic cup during several stages of development, we used it as a reliable marker for the anterior fate in the cup. From previous work [21], we know that the border areas/transition zones are sites of induced collagen IX expression. CollagenIX expression is apparently long-lived at the border and is found in all transition zones that we have examined (example in Figure 6F), while Wnt2b expression is quickly lost (Figure 6I,C). We have demonstrated the ectopic expression of other ciliary body–specific proteins in experimentally created transition zones, but it is not known if they are also induced by Wnt2b. CollagenIX was also induced in the depigmented optic cup lip (Figure 3D). It appears that collagen IX is directly induced by Wnt2b [29], and we hypothesize that lens-derived Wnt2b (Figure 1H) may induce collagen IX in any nonpigmented optic tissue that is in contact with lens. However, as collagen IX was not induced in association with ectopic lenses (Figure 8B-E), this may be a simplistic interpretation.

Although Wnt signaling is involved in many aspects of eye development, it is often integrated with other signaling pathways for various outcomes at different time points [27,49]. Thus, it appears that Wnt signaling, as well as BMP signaling, has a role in specifying RPE fate, but in other contexts these signals are involved in distinct fate decisions [8,22,50–52]. For the specification of tissue in the front of the eye, it has been shown that BMP, FGF, and Wnt signals all have a role [21,48,53,54]. Specification of the anterior optic cup lip has been compared the specification of the neural crest, in terms of borders between the neural and nonneural epithelium, signaling pathways used, and factors present [55]. In the eye, bone morphogenetic proteins 4 and 7 (BMP4 and 7) have dynamic expression patterns during development, but once the optic cup has formed, BMP7 is expressed exclusively in the anterior RPE [14,56]. FGF8 and FGF9 are expressed in the NR domain of the formed optic cup, and strikingly, in FGF9 mutant mouse eyes the border between PE and NPE is shifted internally and the ciliary body is apparently lost [54,56]. Several Wnts are also expressed in the anterior optic cup [24,25,28]. In this report, we forced the expression of FGF and found that Wnt2b was induced at the border between two different epithelial cell types. This is highly reminiscent of recent models for the specification of neural crest (NC) in *Xenopus*, where it is proposed that FGF signaling induces the neural crest indirectly through its activation of a Wnt ligand and its inhibition of the signal [57].

### Model of anterior optic cup development

We propose a model in which the anterior portion of the optic cup is established as the domain between NR and RPE, through the control of Wnt2b expression. Wnt2b is initially induced in the outer layer of the optic cup by the surrounding mesenchyme, and β-catenin signaling is part of the differentiation program for RPE [3,8,52,58]. As development proceeds, the refinement of Wnt2b expression to the optic cup lip establishes the ciliary body, as demonstrated by the induction of collagen IX expression. Refinement is not dependent on the lens, but rather occurs at the border between RPE and nonpigmented tissue.

The model predicts that wherever a border between RPE and NPE exists in the eye, Wnt2b will be expressed and anterior eye fates will be specified; this is what we have shown here. Recent work by Zhang et al. [44] in the developing mouse eye also supports this model. That study documented changes in eye development after the genetic ablation of the prelens ectoderm. Similar to what we report after surgically removing the prelens ectoderm, lensless optic cups formed in the mouse, and the ciliary body and iris were appropriately specified. Interestingly, in their model, small patches of pigmented epithelium form in the central retina of the lensless mouse eyes, and at the borders of those ectopic patches, ectopic ciliary body tissue is specified. Further work is needed to determine whether Wnt2b is ectopically expressed at borders in the mouse as it is in the chick.

During development, borders are established and then elaborated upon, ultimately creating distinct tissues from a field of equivalent cells. The tissues of the eye are not immediately fixed in their cell fates. The use of replication-incompetent retroviruses in this study made it possible to create enduring borders between distinct cell types. With this technique, we were able to demonstrate that Wnt2b is expressed in a pattern that is refined to identify a border, even when that border is ectopically located. We propose a model of anterior eye specification where interacting signals at the border of NPE and RPE tissue induce Wnt2b, which then acts as a signaling center, either alone or with other signals, to organize the other characteristics of the anterior optic cup lip.
